# Nanoparticle Printing for Microfluidic Applications: Bipolar Electrochemistry and Localized Raman Sensing Spots

**DOI:** 10.3390/mi14020453

**Published:** 2023-02-15

**Authors:** Alessia Broccoli, Anke R. Vollertsen, Pauline Roels, Aaike van Vugt, Albert van den Berg, Mathieu Odijk

**Affiliations:** 1 BIOS Lab on a Chip Group, MESA+ Institute for Nanotechnology, Max Planck Center for Complex Fluid Dynamics, University of Twente, 7500 AE Enschede, The Netherlands; 2 Department of Applied Stem Cell Technologies, TechMed Centre, University of Twente, 7500 AE Enschede, The Netherlands; 3 VSPARTICLE B.V., 2612 HL Delft, The Netherlands

**Keywords:** nanoparticle deposition, microfluidic device, bipolar electrodes, surface-enhanced Raman spectroscopy

## Abstract

The local integration of metal nanoparticle films on 3D-structured polydimethylsiloxane (PDMS)-based microfluidic devices is of high importance for applications including electronics, electrochemistry, electrocatalysis, and localized Raman sensing. Conventional processes to locally deposit and pattern metal nanoparticles require multiple steps and shadow masks, or access to cleanroom facilities, and therefore, are relatively imprecise, or time and cost-ineffective. As an alternative, we present an aerosol-based direct-write method, in which patterns of nanoparticles generated via spark ablation are locally printed with sub-mm size and precision inside of microfluidic structures without the use of lithography or other masking methods. As proof of principle, films of Pt or Ag nanoparticles were printed in the chambers of a multiplexed microfluidic device and successfully used for two different applications: Screening electrochemical activity in a high-throughput fashion, and localized sensing of chemicals via surface-enhanced Raman spectroscopy (SERS). The versatility of the approach will enable the generation of functional microfluidic devices for applications that include sensing, high-throughput screening platforms, and microreactors using catalytically driven chemical conversions.

## 1. Introduction

Polydimethylsiloxane (PDMS) is currently the most used material in the fabrication of micro- and nanoscale devices, due to its unique combination of properties including biocompatibility, easy fabrication via soft lithography, gas permeability, transparency, and inexpensiveness, making its use attractive in many fields of science. The integration of metal nanoparticle (NP) films on PDMS microfluidic chips provides flexibility in fabricating functional devices for applications, such as stretchable electronics [1,2], chemical, and biological sensors [3].

However, an economically scalable and reproducible method to obtain and deposit NPs is still a fundamental roadblock. The precise implementation of NP films in specific regions of microfluidic chips is particularly interesting, for example, for the fabrication of devices for high-sensitivity analysis [4], in which the confinement of the detection spot improves the sensing performance, or in the case of multiplexed chips. The direct deposition of NPs in pre-defined patterns or locations on PDMS remains a challenge, as in most cases, a masking layer or a local modification of the surface is needed prior to the NPs deposition on that area.

Conventional methods to perform a local deposition of NPs require vacuum processing and multiple lithographic steps, making the process complex and time-consuming. Metal films can indeed be deposited on PDMS-based substrates via e-beam evaporation or sputtering, which are contamination-free and highly productive methods, and then patterned by a lift-off process [5] or etching. These methods require a protective masking layer during the etching of the unwanted metal portions.

Alternatively, it is possible to directly inject and solidify a conductive solution into PDMS channels, although this technique is limited to metals with a low melting point (<300 °C) and currently allows for the fabrication of structures with cross-sections of 10 µm or larger [6]. Inkjet printing of metal NPs [1,7] is an alternative additive method that enables the deposition and NPs patterning on PDMS without the use of any masks. However, it requires a surface modification of the substrate to optimize the wettability and adhesion of the metal ink on the PDMS, in order to avoid the coalescence of adjacent ink droplets. Moreover, it adds a carrier liquid that might cause unwanted interference with the application.

Metal deposition onto elastomeric substrates can also be obtained via direct metal transfer technology. This method usually requires a cleanroom working condition, as it is based on the fabrication of metal patterns on a rigid substrate (e.g., glass or silicon wafer) before their transfer onto PDMS substrates [8]. The control of the contact time and adhesion forces between the PDMS and the metal patterns plays a crucial role, and an intermediate layer (e.g., Ti [9], Cr [10]) is required to promote adhesion.

Recent advances in the fabrication of ordered arrays include bottom-up approaches based on the self-assembly of NPs, which represents a cost-effective method. Self-assembly involves the evaporation of a suspension on a solid surface [11]. To be able to obtain a local deposition of NPs, the self-assembly process should be performed on regions of the substrate with enhanced adhesion using physical, chemical, or biochemical bonding. Current approaches to fabricate ordered arrays include, for example, template-assisted self-assembly [12], dip-coating self-assembly [13], and directed self-assembly [14], in which patterned substrates are used to facilitate the periodic arrangement of NPs. However, these techniques require multiple steps (nanoparticles synthesis, template preparation and activation of the surface, assembly of the functionalized nanoparticles, and array transfer), and therefore, could potentially be time-consuming and laborious.

An alternative approach to the abovementioned methods is offered by spark ablation, which represents a precise and versatile method to rapidly produce NPs in a gaseous environment. It enables a single-step manufacturing of metal or alloy NPs [15] with different properties [16] and has proven to be a simple, economical, and clean [17] method, as it does not require the use of any chemical precursors and does not generate any hazardous waste. The spark-discharge NP generator consists of a pair of (semi)conductive electrodes separated by a gap and a pulse-forming electrical circuit [18]. The spark discharge between the electrodes generates an aerosol of the electrode material, which is then carried away by a high-purity inert gas (e.g., Ar or N_2_) and can potentially be deposited on any type of substrate (e.g., Silicon [19], Poly(3,4-ethylenedioxythiophene):poly(styrenesulfonate) (PEDOT:PSS) [20], Nafion membranes [21]) by inertial impaction. In addition, size selection of the nanoparticles is possible using an electric field.

To date, this method has not been used to integrate NPs on elastomeric substrates, specifically in PDMS microchips.

**Figure 1:**
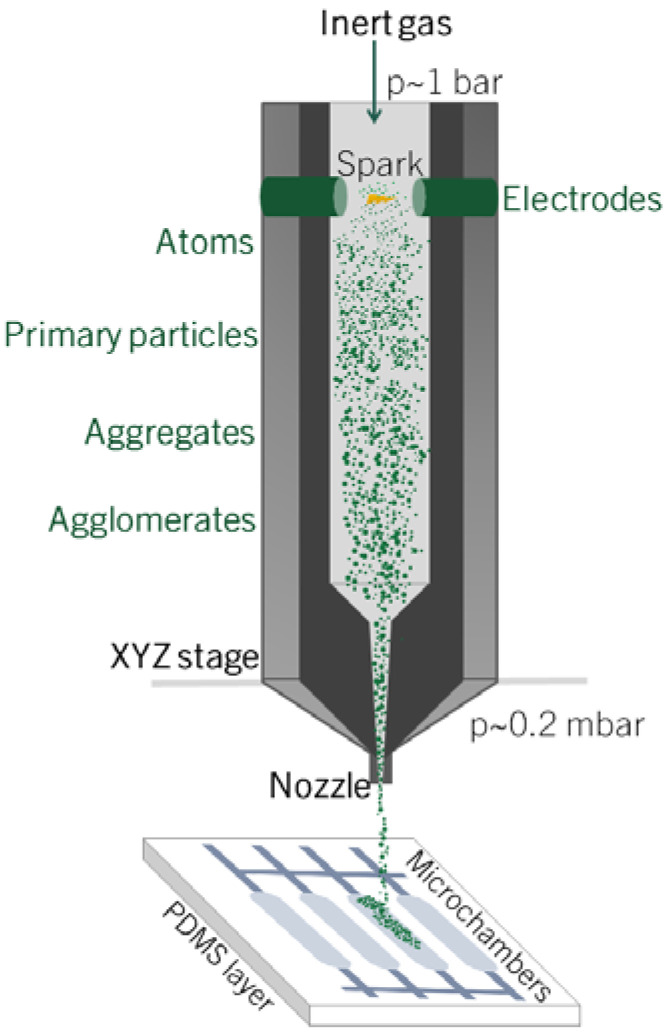
Schematic representation of the nanostructured material printer (VSP-P1 NanoPrinter) used for the deposition of nanoparticle films in the chambers of a multiplexed PDMS microfluidic device.

In this work, we present a novel method to locally deposit NPs generated by spark ablation in the chip chambers of a multiplexed PDMS microfluidic device using a nanomaterial 3D printer from VSParticle (Figure 1). It consists of a particle generator directly connected to a chamber, in which the deposition of the nanoparticles on the substrate takes place through a nozzle via inertial impaction. The fabrication of nanoparticle patterns is performed by placing the substrate on an XYZ stage. The direct control of the deposition area and the fast process rate are among the main advantages of the technique compared to the existing methods. Moreover, the adhesion of the nanoparticles on the substrate is obtained by inertial impaction, without requiring the use of any binder.

We report the effect of the printing parameters (speed of printing and nozzle-substrate distance) on the morphology of the deposited films of nanoparticles. Furthermore, microfluidic devices with integrated Pt and Ag NPs were tested for two different applications:

1. Pt NPs were used to generate potential and pH gradients via bipolar electrochemistry.
2. Ag NPs were used as the surface-enhanced Raman surfaces for the in situ detection of organic molecules, showing the potential and versatility of this new fabrication method.

## 2. Results and Discussion

### 2.1. Nanoparticle Film Characterization

The local deposition of nanoparticles was obtained using the experimental printing setup shown in Figure S1. The focusing nozzle allows for the creation of the desired patterns of NPs on the substrate, in this case, PDMS. The range of feature sizes that can be obtained with the nanoparticle printer depends on the distance between the nozzle and the substrate, as well as on the dimension of the nozzle itself and the speed of printing. The minimum width of the printed features mostly depends on the nozzle throat, which is 100 µm in our case. We first studied the effect of the main printing parameters, the distance between the nozzle of the printer and the substrate, and the speed of printing. A first observation is that we see the formation of metal films, formed by agglomerates of nanoparticles unless really fast printing speeds are used. The distance between the nozzle and the substrate affects the morphology of the metal films, as shown in Table 1 with Pt and Ag NPs printed with nozzle-substrate (N-S) distances of 200 and 500 µm. The nozzle distance defines the width of the lines, as the closer the nozzle is to the substrate, the lower the expansion of the gas containing the NPs. Moreover, the speed of printing influences the width of the lines, as well as their thickness. The slower deposition speed corresponds to thicker lines, due to the higher amount of NPs focused on the substrate per unit of time.

Therefore, it is possible to control the morphology of the desired 3D pattern by controlling the printing parameters. The impact of the NPs beam on the elastomeric surface leads to local heating and expansion of the PDMS surface during the deposition [22], resulting in the formation of cracks on the metal films. As shown in the pictures in Table 1a, in the case of Pt, the cracks are more evident on the lines printed with the fastest speed. The slower printing speeds result in a thick and compact layer of NPs (Figure S2), which tend to better dissipate the heat involved in the printing process. In contrast, Ag printed with slower speeds show a larger amount of cracks (Table 1b. We hypothesize this is due to the contraction of the silver film under residual internal stress. For our experiments, we used printing parameters that did not lead to crack formation. Additionally, we noticed that increasing the working distance can cause a random and low density deposition of nanoparticles and agglomerates outside of the printed line’s region.

The nanoparticles generated via spark ablation are characterized by a diameter between 0 and 20 nm [23] and tend to agglomerate and form clusters due to collision and coalescence, which may occur on the way to the nozzle, as well as directly on the substrate, due to the clean surface of the particles and the favorable metal-metal contact [23].

Before the deposition of the nanoparticles, the PDMS surfaces were plasma treated. During this process, the PDMS is exposed to the oxygen plasma, which generates silanol functional groups and increases the surface roughness of the material, enhancing the adhesion strength of the metal layer to the elastomeric surface [24]. Stability tests to check the adhesion of the printed NPs were performed on-chip, depending on the applications that will be discussed in the following sections. To check the stability of the NP films in the chambers, water was flushed in the chip for 48 h. Samples were collected at the outlet periodically and investigated with UV/Vis analysis, which did not reveal the presence of Pt/Ag NPs, and thus confirmed their stability on the PDMS substrate. Tape tests were also performed on NPs printed on flat PDMS layers using a scotch transparent tape 3 M (Section 6 in Supplementary Materials), and the grade of adhesion was evaluated by optical analysis, inspecting any detachment of the metal film from the substrate. Moreover, in this case, no Pt/Ag was released during the adhesion tests.

### 2.2. Application I: Bipolar Electrodes in a Multiplexed Microfluidic Chip

The possibility of locally printing metal NPs facilitates the integration of electrodes in microfluidic devices, which represent essential components for a broad range of applications including electrokinetic transportation or electrochemical detection. Particularly, electrochemical generation and control of gradients are widely used to create both static or dynamic solutions and surface gradients, as well as to control pH and O_2_ concentration [25]. These gradients can be used to reproduce cellular environments and play an important role in the study of cell adhesion [26] or the control of protein conformation [27]. Moreover, gradients are employed in the high-throughput generation and screening of materials [28], such as catalysts, or in the development of sensing platforms [29].

In this section, we propose an attractive alternative method to the current screening platforms, consisting of an array of individually addressable chambers embedding 3D-printed Pt electrodes, which enable the generation and comparison of potential gradients over the electrodes.

**Figure 2:**
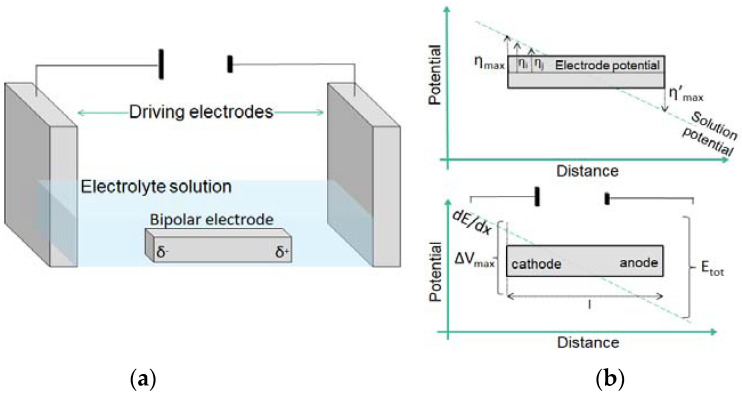
(**a**) Schematic illustration of a bipolar electrode setup. (**b**) Schematic of the magnitude of the interfacial potential difference between the BPE and the solution. The magnitude of the overpotentials varies along the length of the electrode, assuming the highest values at its extremities.

A convenient configuration to integrate arrays of electrodes in a microfluidic device, thus increasing the processing throughput, relies on the use of bipolar electrodes (BPEs). A BPE consists of an isolated patch or strip of conductive material immersed in an electrolyte solution (Figure 2), between two external electrodes to generate an E-field. The main advantage of their use is the possibility to maintain control over single or multiple electrodes without direct electrical contact [30], facilitating their integration and parallelization in a chip. The polarization of a BPE derives from the interfacial potential difference between the solution and the surface of the electrode. This can be used to drive Faradaic reactions [31]. As shown in Figure 2b, the voltage difference is highest at the edges of the electrode that act as cathode and anode simultaneously.

When a sufficient driving potential $E_{tot}\;$is applied in a channel, the fraction of $E_{tot}$ dropped over the BPE depends on the distance between the driving electrodes ($l_{channel}$) and the length of the electrode itself ($l_{electrode}$), and can be estimated as follows [32]:

(1) $$\;\;\;\;\;\;\;\Delta E_{elec}=E_{tot}\frac{l_{electrode}}{l_{channel}}$$

It is also important to underline that since the polarization varies along the conducting BPE, a potential gradient is formed along the bipolar electrode, which allows for the screening of different thermodynamic conditions in one experiment [33].

The understanding of electrokinetic and electrochemical dynamics in microfluidic-based bipolar systems [34] led to their use for a wide range of applications, including the simultaneous enrichment and separation of analytes [35,36], as recently demonstrated with microplastics [37]. Arrays of BPEs have also been used for sensing, relying on electrochemiluminescence [38] or electrodissolution of the electrodes [39] themselves, or as screening platforms to study the activity of metal electrocatalysts [40].

**Figure 3:**
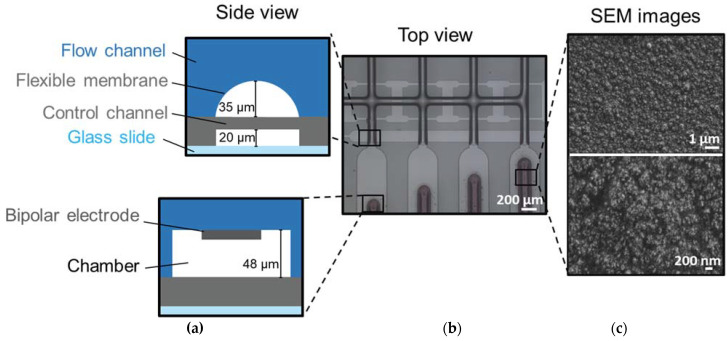
(**a**) Schematic cross-section and (**b**) optical image of the multiplexed PDMS microfluidic device. Pt NPs are printed in the chip chambers (flow layer). (**c**) SEM images of the morphology of Pt NPs patterns deposited on the PDMS substrate. Voltage/current settings used for the deposition, V = 1.3 kV and I = 10 mA, respectively, speed of printing of 10 µm/s, and nozzle-substrate distance of 500 µm. Film thickness of ~0.4 µm.

Pt electrodes were 3D-printed in a PDMS microfluidic device with four independently addressable chambers and used as BPEs, which enable the generation and comparison of potential gradients. Figure 3c shows an example of the typical morphology of the Pt film on the PDMS substrate, composed of a dense and rough layer of nanoparticles with a mean particle size of 33.7 ± 4.2 nm and larger aggregates of hundreds of nanometers. 

Each chamber results in a confined environment, where the produced gradients can be tuned and monitored without affecting the activity of the nearby electrodes.

Figure 3 illustrates the microfluidic device consisting of two PDMS layers and a glass slide. We have previously reported a similar device for high-throughput cell culturing [41] and stem cell differentiation [42]. The top layer contains the fluidic channels and chambers, where the Pt electrodes are confined. The control channels and valves are placed on the bottom layer, which is directly attached to a glass slide. Pressurizing the control line will make the membrane bend into the flow channels, thereby blocking the flow as well as the electrical (ionic) current.

The operation of the microfluidic device was demonstrated by performing water electrolysis as a model reaction. When the externally applied electric field is sufficiently large (i.e., higher than the difference in the formal potentials for the two redox reactions [43]), the electrode is sufficiently polarized to drive simultaneously redox reactions at its poles, which are given by [44]:

(2) $$O_{2}+4H^{+}+4e^{-}⇋2H_{2}O\;\mathrm{water}\mathrm{oxidation}\mathrm{at}\mathrm{acidic}\mathrm{pH}$$

(3) $$O_{2}+2H_{2}O+4e^{-}⇋4OH^{-}\;\mathrm{water}\mathrm{oxidation}\mathrm{at}\mathrm{basic}\mathrm{pH}$$

(4) $$2H^{+}+2e^{-}⇋H_{2}\;\mathrm{water}\mathrm{reduction}\mathrm{at}\mathrm{acidic}\mathrm{pH}$$

(5) $$2H_{2}O+2e^{-}⇋H_{2}+2OH^{-}\;\mathrm{water}\mathrm{reduction}\mathrm{at}\mathrm{basic}\mathrm{pH}$$

The listed reactions lead to changes in the pH and conductivity of the solution at the BPE poles, due to the consumption or generation of H^+^ and OH^−^. Specifically, the O_2_ generation at the anodic pole of the BPE decreases the pH of the solution with OH^−^ consumption/H^+^ production; while the H_2_ generation at the cathodic pole increases the pH due to the H^+^ consumption/OH^−^ production. To visualize the induced electric field on the BPE and follow the water electrolysis reaction, we monitored the changes in the fluorescence intensity of the pH-sensitive Fluorescein sodium salt (FL). FL exists in different forms (FL^+^, FL^0^, FL^−^, FL^2−^); therefore, its fluorescence properties strongly depend on the pH of the electrolyte. Particularly, it exists in its cationic form at pH < 2 and undergoes from FL^−1/−2^ to FL^0^ at pH < 4. FL shows a strong fluorescence at pH above 6.5 [45]. During water electrolysis, a dynamic pH gradient is formed along the electrodes due to the aforementioned faradaic reactions, and thus it affects the charge and the fluorescence intensity of the tracer molecule FL. During water electrolysis, the O_2_ generation and consequent decrease in the pH cause quenching of the fluorescence intensity, while the increase in the pH should increase/maintain the intensity value as constant, depending on the starting pH of the solution. The pK_a_ value of the FL is close to the pH of the electrolyte solution used in the experiments; therefore, there is a quick change in the FL net charge and fluorescence intensity according to the induced pH profile [46] (Figure S3).

**Figure 4:**
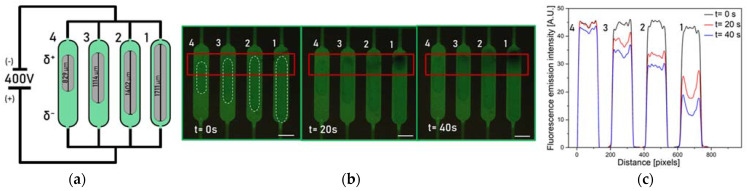
(**a**) Schematic (not to scale) of the experiments performed using the Pt electrodes in the chambers simultaneously. (**b**) Fluorescence images showing the activity of BPEs used simultaneously upon the application of an external driving potential $E_{tot}\;$= 400 V. (**c**) Fluorescence emission intensity profile at the anodic poles of the electrodes. The change in fluorescence intensity reflects the decrease in the pH of the solution, due to the O_2_ production via water splitting. The available overpotential for O_2_ production is proportional to the length of the electrodes. Scale bars: 350 µm.

As already mentioned, the fraction of $E_{tot}\;$ dropped over each BPE, which depends on the ratio between the length of the electrode and the microchannel (Equation (1). Figure 4 shows the microfluidic device with four Pt BPEs obtained using the same printing parameters (N-S = 500 µm and speed of printing 10 µm/s), and thus have the same morphology (thickness, particle size), but different lengths. The chambers containing the BPEs are filled with an aqueous solution of 100 µM FL and 1.0 mM sodium phosphate buffer at pH 7.2. An external electric field was applied at two Pt wires used as driving electrodes, inserted into the inlet and outlet of the chip, and connected to a power supply to introduce a potential difference of 400 V between the electrodes (corresponding to an electric field of ~12 kV/m). The complete design of the chip used for the experiments and its equivalent circuit are shown in Figure S4.

These high values of external potential are needed to visualize the O_2_ and H_2_ generations at the poles of the electrodes, since the bipolar configuration is far from standard conditions (at which $E^{0}=1.23\;V)$. The length of the channel (3 cm), corresponding to the distance between the driving electrodes, is an order of magnitude higher than the dimension of the bipolar electrodes (~1 mm) in the chambers.

The application of a potential difference between the driving electrodes generates an electric field, which produces a linear change in the potential across the solution (Figure 2b), while the floating electrode is itself equipotential. The presence of a potential gradient in the solution results in the presence of a continuum of different driving forces along the electrode for electrochemical reactions, with a maximum value at the edges. In the experiments, the length of the channel (3 cm) is the same for each electrode, while their lengths are different (from chamber 1 to chamber 4, the lengths of the BPEs are 829, 1114, 1402, 1711 µm). This results in higher values of $\Delta E_{elec}$ on longer electrodes (Equation (1)), corresponding to a more pronounced quenching of the fluorophore as can be seen in Figure 4c. The fluorescence intensity of the dye drops starting from the anodic pole of the electrodes, due to the acidification of the solution. Moreover, the different lengths of the electrodes result in different portions of substrates sufficiently polarized to drive the water oxidation; indeed, the longer electrode shows a larger active portion toward the O_2_ generation. With these considerations, it is possible to design the electrodes, for example, according to the desired potential drop or screen the effect of different potentials on the reaction of interest.

**Figure 5:**
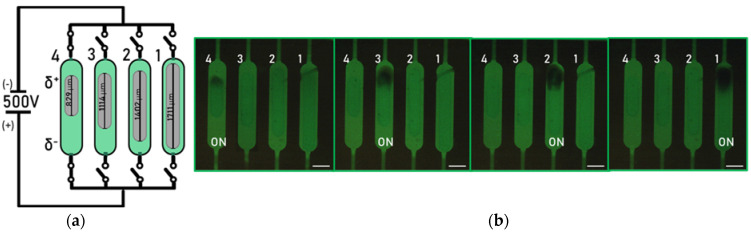
(**a**) Schematic (not to scale) of the experiments performed using the Pt electrodes in the chambers independently. The PDMS valves are represented by switches. The open configuration of the valve corresponds to a closed switch, thus the current can go through the chamber. (**b**) Fluorescence micrographs showing the activity of the BPEs used individually upon the application of an external driving potential $E_{tot}\;$= 500 V. The change in the fluorescence intensity reflects the decrease in the pH of the solution, due to the O_2_ production via water electrolysis reaction. The electrodes are used independently thanks to the presence of PDMS valves, which can act as insulators by closing the conductive path through the channels. Scale bars: 350 µm.

One of the advantages of the proposed device is the confinement of the electrodes in parallel PDMS chambers, by which is it possible to avoid the influence of the electrodes nearby. The device allows for the use of each electrode individually (Figure 5), thanks to the presence of pneumatically actuated valves in the ‘’push-up’’ configuration, which can electrically insulate the electrodes in the path not in use.

### 2.3. Application II: SERS Structures in a Microfluidic Device for Organic Molecule Detection

At present, surface-enhanced raman spectroscopy (SERS) is an established and powerful analytical technique for the detection of low concentrated samples, and it is based on the amplification of the Raman signal of molecules adsorbed on nanostructured metallic surfaces. The improvement of the signal can be attributed to both electromagnetic and chemical enhancement [47], with the first being the dominant contributor. The electromagnetic enhancement mechanism is related to the amplification of the light by the excitation of localized surface plasmon resonances (LSPRs), while the chemical enhancement involves a charge-transfer mechanism [48], in which the excitation wavelength is resonant with the metal-molecule charge-transfer electronic states. Noble metals, such as gold, silver, or copper, are extensively used as SERS substrates, as all of them have LSPRs that cover most of the visible and near-infrared wavelength range, thus enhancing the measurement of the Raman signal. Among them, Ag exhibits the best optical absorption and scattering properties, and has been widely employed in chemical and biomedical detection [49,50]. The integration of Ag nanoparticles in a microfluidic device results in a promising approach, as it can enable the static and dynamic liquid measurement of the samples, resulting in an in situ, real-time detection system [51]. The fabrication of SERS substrate in PDMS microfluidic devices is usually obtained from the reduction in Ag precursors injected in the channels [52], thus requiring precise control of the flow and heating conditions to obtain the homogeneous deposition of the metal along the channel. The spark ablation method and the use of a 3D nanoparticle printer provide an alternative, innovative, and convenient approach for the generation of local SERS-active patterns, as it represents a one-step process to obtain Ag NPs substrate in a microfluidic channel. To be able to study the possible Raman enhancement given by the Ag substrate, the NPs were deposited in the chip chambers of the microfluidic device reported in Section 2.2 with a printing speed of 60 µm/s and nozzle-substrate distance of 500 µm. Table S1 reports the effects of different printing speeds and N-S distances over the Ag NPs printed on PDMS layers.

**Figure 6:**
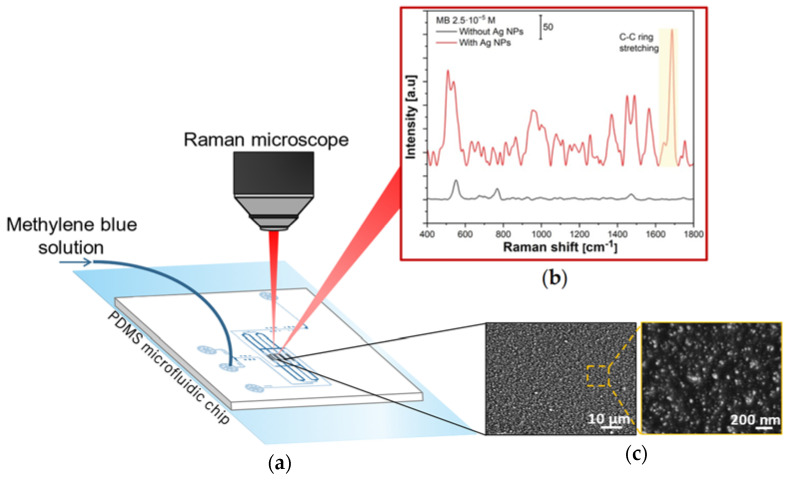
(**a**) Schematic representation of the acquisition of the Raman signals from a PDMS chip with Ag NPs deposited in its chambers. (**b**) Raman spectra of MB obtained by focusing the laser beam on areas of the chip chambers without (in black) and with Ag NPs (in red) deposited on the PDMS. The black curve only shows the PDMS peaks, while the red curve represents MB peaks. In both cases, the fluorescence background was subtracted. The highlighted peak refers to C-C stretching and it was used for the enhancement calculation. The additional MB characteristic peaks and vibrational modes are reported in Figure S5. (**c**) SEM images of Ag NPs deposited on PDMS. Voltage/current settings used for the deposition, V = 1.3 kV and I = 10 mA, respectively, speed of printing of 60 µm/s, and nozzle-substrate distance of 500 µm.

The performance of a SERS substrate is influenced by the size, density, and morphology of metal NPs, due to the influence of these factors upon the electromagnetic field. Particularly, the electromagnetic field can be highly enhanced in the presence of nanogaps or hot spots between adjacent NPs [53], thus increasing the SERS intensity. On the other hand, when the NPs size and fill factor increase, the absorption of light is less effective and the absence of nanogaps decreases the electromagnetic enhancement. Moreover, aggregates of NPs of hundreds of nanometers have a low surface area compared to bare NPs, and thus lower adsorption of the target molecule. Figure 6c shows a film of Ag NPs deposited on the PDMS surface, mainly consisting of particles with diameters ranging from 0–35 nm and some clusters of 40–50 nm, resulting in a suitable substrate for SERS.

To evaluate the SERS performance of the Ag NPs, a solution of methylene blue (MB) of 2.5·10^−5^ M was used as a probe molecule. The characteristic Raman peaks of MB are shown in Figure S5, with the most intense highlighted in Figure 6b at 1623 cm^−1^ corresponding to C-C ring stretching [54].

Before the detection of MB on the Ag layer, the laser beam was focused on the bare PDMS and its Raman spectrum was collected to be able to distinguish its peaks (Figure S6) from the ones of the target molecule.

To investigate the reproducibility of the substrate, five points were randomly selected on the Ag substrate to collect the signal of the organic molecule (Figure S7). A comparison between the Raman spectra of MB obtained by focusing the laser in the chip chambers on the bare PDMS and areas with the Ag nanoparticles deposited on the PDMS is reported in Figure 6b. Moreover, the activity of the AgNPs toward the Raman enhancement was checked using freshly prepared devices and with chips stored at ambient conditions for a week, and the results obtained showed the stability of the signal (Figure S8). For the experiments, the aqueous solution of MB was injected into the chip chambers and left in contact with the substrate for 1 h, and the detection of the liquid was performed by focusing on the sensing spots. The Raman signals of the MB 2.5·10^−5^ M were not detectable on the bare PDMS, as it only exhibits PDMS characteristic peaks (Figure 6b, black curve). On the other hand, the SERS signals of MB could be detected from the Ag substrate. In this case, the characteristic Raman peaks of MB results shifted by ~60 cm^−1^ compared to the typical MB spectra reported in the literature, suggesting chemisorption of the sample molecules on the Ag structure [55].

To quantify the SERS enhancement, we referred to the analytical enhancement factor (AEF) [56], which is commonly used to relate the signal intensity to the analyte concentration. It provides information about the signal gain that the SERS substrate produces, in comparison to a reference Raman experiment with the same analyte and under the same experimental conditions. The analytical enhancement factor (AEF) was evaluated by taking into account the most intense signal from the methylene blue spectrum (1623 cm^−1^) and estimated using the following equation:

(6) $$AEF=\frac{C_{Raman}}{C_{SERS}}\cdot \frac{I_{SERS}}{I_{Raman}}$$

$C_{Raman}$ and *C_SERS_* represent the concentrations of the aqueous MB solutions used for the Raman (0.11 M) and SERS (2.5·10^−5^ M) measurements, while $I_{Raman}$ and $I_{SERS}$ are the intensities of the corresponding peaks representative of C-C ring stretching. A value of (1.7 ± 0.3)·10^5^ was obtained as AEF, comparable to a typical average EFs value reported in the literature [56] for Ag structures.

## 3. Conclusions and Outlook

In this work, we proposed a new, simple, and one-step process to locally print patterns of nanoparticles on PDMS. The nanoparticles are produced by spark ablation and directly deposited and patterned on the elastomeric substrate using a 3D printer, which allows for the control of the deposition area, resulting in particular interest for the integration of metal NP films in microfluidic devices.

The variation of printing parameters, such as the speed of printing and the distance between the nozzle of the 3D printer and the substrate, influence the morphology of the deposited layers. High speed of printing lead to a thin film of nanoparticles, while the distance between the nozzle of the 3D printer and the substrate influences the width of the lines. Therefore, it is possible to obtain full control over the desired NPs pattern.

We integrated Pt and Ag nanoparticles in a PDMS multiplexed microfluidic device used respectively to generate potential and pH gradients and as SERS sensor. In the first case, we confirmed the conductivity of the printed lines and obtained a device with parallel electrodes, which can be used simultaneously and individually. The platform can be used for sensing or the high-throughput generation and screening of materials. The electrodes can indeed be used to control pH and O_2_ concentration, as well as to create both static or dynamic solutions and surface gradients.

In the second case, Ag films were used as SERS substrate and allowed for the detection of a solution of MB 2.5·10^−5^ M, with an AEF ≈ 1.7·10^5^. This result can be further improved by screening the enhancement of the electric field of NPs obtained with different printing parameters or materials. The integration of SERS with microfluidics enables both the static and dynamic liquid measurements, offering more reliable and reproducible results than the static solid ones.

The results show the versatility of the method and its high potential for the functionalization of microfluidic structures. Moreover, it is possible to deposit metal alloys using electrodes of different materials in spark ablation, or metal oxides using air as a carrier gas, which further increases the application fields. Preliminary experiments performed with TiO_2_ show its possible use as photocatalyst.

The method allows for the easy integration of metal nanoparticles in specific regions of microfluidic devices for the fabrication of functional devices for applications, including sensing and screening platforms and microreactors.

## 4. Materials and methods

### 4.1. Nanoparticle Generation and Printing

Pt and Ag NPs were produced and deposited on the PDMS substrates using a commercial particle generator (VSParticle-G1) and an aerosol printer (VSP-P1 NanoPrinter). The generation of NPs is obtained via spark ablation, which consists of an evaporation-condensation mechanism. The energy of the spark plasma leads to the evaporation and sublimation of a portion of the electrodes, and then, the metal vapor is quenched and condensated via adiabatic expansion and mixing with the carrier gas [57], producing particles with diameters in the range of 0–20 nm. Subsequently, the particles tend to interact and agglomerate.

The particle generator was equipped with 2x Pt (99.9%) or Ag (99.9%) electrodes and 1 slm Ar (99.9%) was used as carrier gas. The voltage and current settings for the spark discharge were 1.3 kV and 10 mA. The deposition unit consists of a prototype nanomaterial printer, composed of a vacuum chamber (pressure in the chamber is 0.0002 bar) directly connected to the output of the particle generator, equipped with a nozzle (throat diameter 0.1 mm) for focusing and printing the beam of NPs on the substrate. The spot size of the nanoparticle beam depends on the nozzle-substrate distance, which can be adjusted between 200 and 1000 µm. To be able to see and control the deposition area, a camera is placed behind the nozzle. The PDMS substrate is placed and fixed perpendicularly to the particle beam on a motorized XYZ stage. Therefore, NPs were deposited by inertial impaction of the aerosol directly in the PDMS chip chambers.

### 4.2. Characterization of the Nanoparticle Films

NPs film thickness was measured by a Dektak Stylus optical profilometer and each line was checked on three different points along the length, in order to verify the uniformity of the printing method and evaluate the standard deviation of the value. The SEM measurements were performed using a Zeiss MERLIN HR-SEM.

### 4.3. Chip Fabrication

The microfluidic devices consisted of PDMS chips with four independently addressable chambers. Their design and microfabrication are based on previous work [41]. Briefly, they were obtained by standard photolithography: SU8 (MicroChem, Round Rock, TX, USA) was used for the control layer wafers with 20 µm of high channels, while for the flow layer wafers, SU8 was used to first obtain channels with rectangular sections (~48 µm high) in the areas without valves, and AZ40XT (MicroChemicals, Ulm, Germany) was used to create channels of ~35 µm high with a rounded profile. In the area with valves, the rounded profile of the flow channels is needed to ensure their correct closing without leakage.

The chips were obtained by multilayer soft lithography. PDMS (RTV615, Permacol, The Netherlands) base and curing agent were mixed to obtain the flow (7:1 *w/w* base to curing agent) and control layers (20:1 *w/w* base to curing agent). The PDMS was degassed for about 2 h.

A ~30 µm thick layer of PDMS was obtained on the control layer by spin coating, while the flow layer was obtained by pouring the PDMS on the mold. Both wafers were cured at 60 °C for 45 min. Once cooled, the PDMS flow layer was cut from the wafer and the inlets and outlets were punched with a 1 mm hole puncher. Then, the flow layer was plasma treated and used as a substrate for nanoparticle printing. After the deposition of the NPs on the flow layer, it was aligned on the control wafer using a stereomicroscope (Olympus), and the layers were cured overnight at 60 °C.

The chip was cut from the wafer and the control inlets were punched out with a 0.75 mm biopsy puncher. Finally, the chip was bonded to a microscope glass slide using a plasma cleaner (model CUTE, Femto Science, South Korea).

The valves of the microfluidic chip were driven by pneumatic actuation. The channels of the control layer were filled with water and pressurized with air (1.5 bar) by solenoid valves (Festo, The Netherlands), which can be controlled via a custom LabView program.

### 4.4. Bipolar Experiments

For the bipolar electrode experiments, the driving electrodes consisted of Pt wires (99.9%, Sigma-Aldrich, Taufkirchen, Germany) located at the inlet and outlet of the chip. They were connected to a power supply (2401 Keithley Source Meter) via external contacts to apply a potential difference between the electrodes. The voltage/current settings used for the deposition of bipolar electrodes are V = 1.3 kV and I = 10 mA, respectively, speed of printing of 10 µm/s, and nozzle-substrate distance of 500 µm. Before the alignment with the control layer, the resistance of the Pt electrodes was measured using a multimeter (Fluke 179), providing a conductivity value ≈ 5.6·10^6^ S/m.

The fluorescent experiments were carried out with a mixture of 100 µM Fluorescein sodium salt (FL) (Sigma-Aldrich) and 1.0 mM sodium phosphate buffer at pH 7.2. The solutions were prepared using deionized water (<18.2 MΩ cm, PURELAB flex). All solutions were maintained away from light by covering the vials with aluminum foil when not in use. For the image acquisition, a microscope Olympus IX51 and a Grasshopper^®^3 (FLIR, US) color camera were used with a pE300^ultra^ LED illumination system (CoolLED, Andover, UK).

### 4.5. SERS Measurements

SERS measurements were carried out using a confocal Raman microscope (WITec Alpha300R, Ulm, Germany). An He-Ne laser with a wavelength of 633 nm was used and focused on the substrate using a lens with a 100× magnification, and a numerical aperture of 0.9. The integration time was 1 s, and the power applied was 10 mW. Higher power led to a massive heating of the PDMS and consequent detachment of the Ag substrate. The methylene blue (Sigma-Aldrich) solutions (2.5·10^−5^ and 0.11 M) were prepared using DI water.

## Figures and Tables

**Table 1:** Optical images and height and width values of Pt (**a**) and Ag (**b**) lines printed on PDMS with different N-S distances (200 and 500 µm) and speed of printing (10–80 µm/s).

(a)						(b)					
Pt NPs						Ag NPs					
**Nozzle-substrate distance** **200 µm**	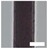	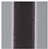	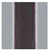	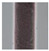	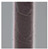	**Nozzle-substrate distance** **200 µm**	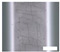	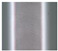	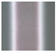	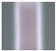	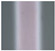
**Speed of printing [µm/s]**	10	20	40	60	80	**Speed of printing [µm/s]**	10	20	40	60	80
**Height [µm]**	0.53 ± 0.04	0.43 ± 0.01	0.34 ± 0.01	0.280 ± 0.003	0.250 ± 0.006	**Height [µm]**	0.79 ± 0.01	0.500 ± 0.004	0.270 ± 0.005	0.170 ± 0.006	0.15 ± 0.01
**Width [µm]**	143.42 ± 0.56	134.09 ± 1.08	126.22 ± 0.01	121.820 ± 0.003	118.73 ± 0.45	**Width [µm]**	238.26 ± 0.55	198.49 ± 1.12	173.36 ± 0.03	151.07 ± 2.82	135.72 ± 1.05
**Nozzle-substrate distance** **500 µm**	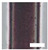	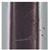	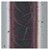	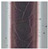	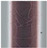	**Nozzle-substrate distance** **500 µm**	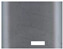	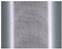	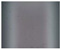	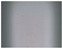	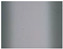
**Speed of printing [µm/s]**	10	20	40	60	80	**Speed of printing [µm/s]**	10	20	40	60	80
**Height [µm]**	0.44 ± 0.02	0.32 ± 0.05	0.24 ± 0.01	0.22 ± 0.02	0.20 ± 0.02	**Height [µm]**	0.56 ± 0.01	0.41 ± 0.06	0.24 ± 0.04	0.15 ± 0.02	0.11 ± 0.03
**Width [µm]**	197.59 ± 0.07	193.17 ± 0.02	184.21 ± 0.45	175.58 ± 0.06	171.04 ± 0.44	**Width [µm]**	317.82 ± 1.16	275.72 ± 3.34	222.93 ± 4.81	204.73 ± 3.07	201 ± 5

For each speed of printing, 3 lines of 1 mm were printed on PDMS substrates and the standard deviation of width and thickness was evaluated on 3 different points along each line. Slower deposition speeds result in thicker lines, as a higher amount of NPs is focused on the substrate per unit of time. All lines were printed using a nozzle throat of 100 µm, and the voltage and the current setting for the spark discharge were 1.3 kV and 10 mA respectively. Scale bars 100 µm.

## Data Availability

The data presented in this study are available on request from the corresponding author.

## Supplementary Materials

The following supporting information can be downloaded at: https://www.mdpi.com/article/10.3390/mi14020453/s1, Figure S1: Equipment used for the nanoparticle deposition; Figure S2: SEM images of a Pt electrode on PDMS; Table S1: SEM images of Ag nanoparticle patterns on PDMS; Figure S3: Fluorescence emission intensity of Fluorescein sodium salt at different pH; Figure S4: Equivalent circuit of the microfluidic device; Figure S5: Raman spectrum of MB 0.11 M on PDMS; Figure S6: Raman spectrum of bare PDMS; Figure S7: Raman spectra of 2.5·10^−5^ M MB; Figure S8: Raman spectra of 2.5·10^−5^ M MB using a device with freshly printed Ag NPs patterns and with a device stored at ambient conditions for a week; Video Section 6: Tape test.
