# Supplementary material for: Nanoparticle Printing for Microfluidic Applications: Bipolar Electrochemistry and Localized Raman Sensing Spots
Source: Micromachines (Basel). 2023 Feb 15;14(2):453. doi: 10.3390/mi14020453 (PMC9967861; doi:10.3390/mi14020453)
Supplement: Supplementary file 1 [file micromachines-14-00453-s001.zip › micromachines-2208311-supplementary.pdf]

Supplementary Information

Nanoparticle Printing for Microfluidic Applications: Bipolar Electrochemistry and Localized Raman Sensing Spots

Alessia Broccoli [1,*], Anke R. Vollertsen [2], Pauline Roels [3], Aaike van Vugt [3], Albert van den Berg [1] and Mathieu Odijk [1]

[1] BIOS Lab on a Chip Group, MESA+ Institute for Nanotechnology, Max Planck Center for Complex Fluid Dynamics, University of Twente, 7500 AE Enschede, The Netherlands
[2] Department of Applied Stem Cell Technologies, TechMed Centre, University of Twente, 7500 AE Enschede, The Netherlands
[3] VSPARTICLE B.V., 2612 HL Delft, The Netherlands
* Correspondence: a.broccoli@utwente.nl

Table of contents

1. Equipment used for the nanoparticle deposition
2. SEM images
3. Calibration curve of Fluorescein sodium salt
4. Equivalent circuit of the microfluidic device
5. Raman spectra
6. Tape test

1. Equipment used for the nanoparticle deposition

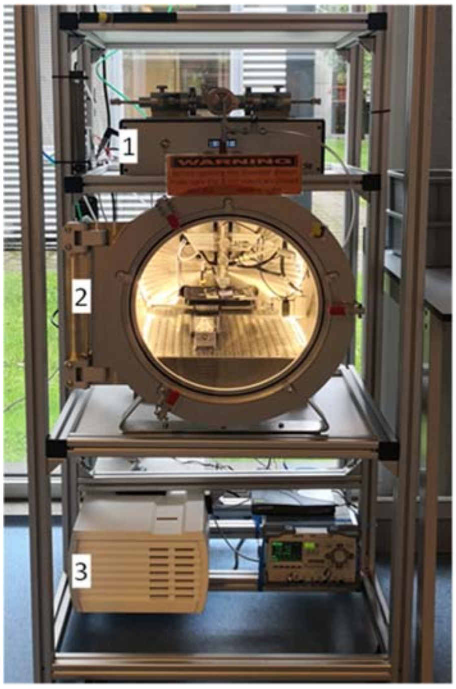

Figure S1: Picture of the prototype VSP-P1 nanostructured material printer setup. 1. VSP-G1 nanoparticle generator. 2. Vacuum chamber with deposition unit 3. Vacuum pump.

2. SEM images

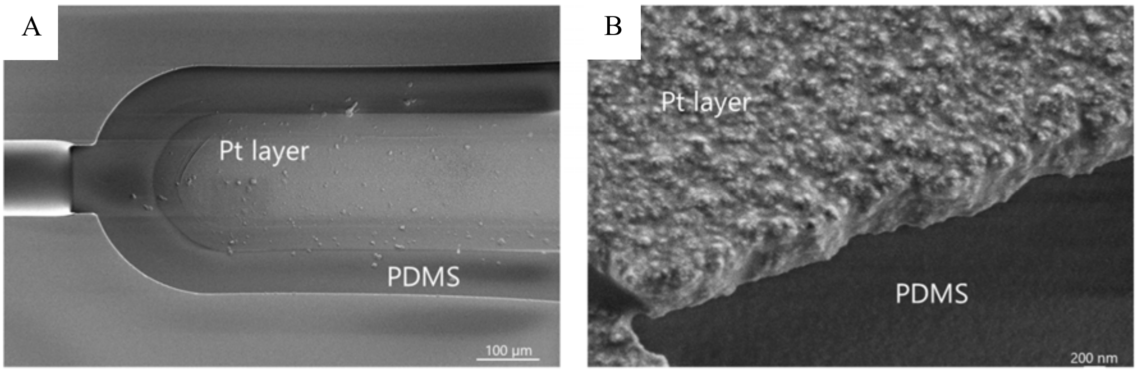

Figure S2: SEM image of A) top view and B)cross-section of Pt NP layer printed on a PDMS substrate at 10 µm/s, with a nozzle-substrate distance of 500 µm. The printing parameters define the morphology of the deposited material, which results in the formation of a dense layer of NPs in the case of printing speeds of 10 µm/s.

Table S1: SEM images and average of the roughness profile of Ag NPs patterns deposited on PDMS at different printing speeds (10- 80 µm/min) using nozzle- substrate distances of (A) 200 µm (B) and 500 µm.

A

Nozzle-substrate distance 200 µm

SEM images	Speed of printing [µm/min]	Mean particle size [nm]	Standard deviation	Roughness average [nm]
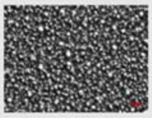	10	30.5	± 2.14	14.8
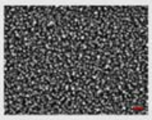	20	29.9	± 2.9	14.8
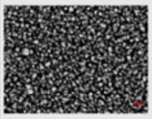	40	29.5	± 3.8	14.5
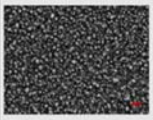	60	28.6	± 1.7	14.3
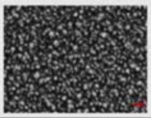	80	28.0	± 1.4	13.1

B

Nozzle-substrate distance 500 µm

SEM images	Speed of printing [µm/min]	Mean particle size [nm]	Standard deviation	Roughness average [nm]
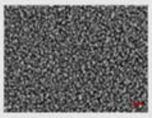	10	38.0	± 1.2	15.8
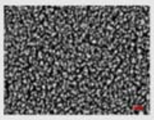	20	37.9	± 0.8	15.4
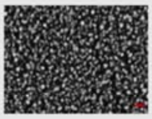	40	33.0	± 1.6	15.2
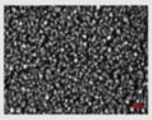	60	33.1	1.5	14.9
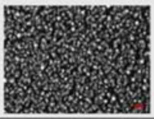	80	33.0	± 1.5	14.4

With slower printing speeds, both the mean particle size and mean surface roughness values are higher compared to the results obtained with slower printing speeds. This can be explained by the higher amount of NPs deposited on the substrate per unit of time, resulting in more collision and coalescence events between NPs. Scale bars 100 nm.

3. Calibration curve

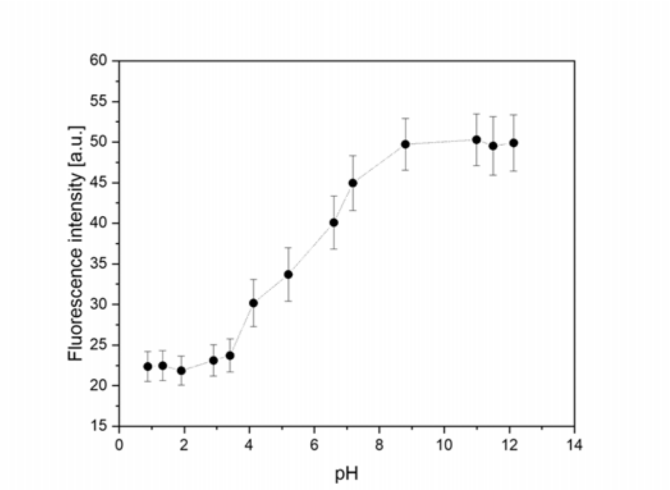

Figure S3: Effect of pH on the fluorescence emission intensity of solutions containing 100 µM fluorescein and 1.0 mM sodium phosphate buffer. The pH of the solutions varies from 0.9 to 12.1. Error bars correspond to the standard deviation of the average fluorescence emission intensity of the solutions in the 4 chambers of the device.

4. Equivalent circuit of the microfluidic device

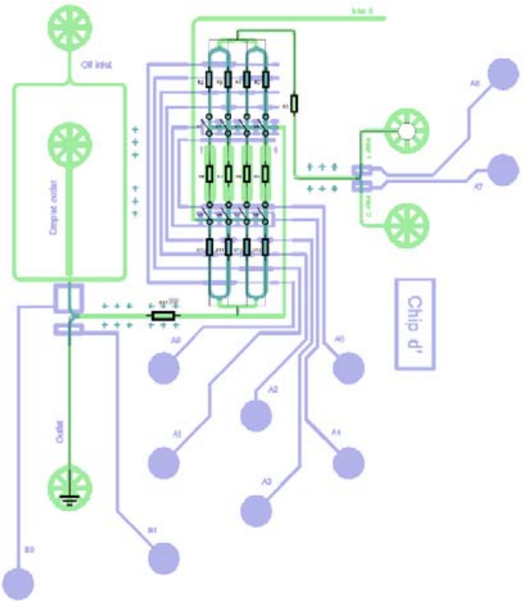

Figure S4: Design and equivalent circuit of the microfluidic device used for the bipolar experiments.

5. Raman spectra

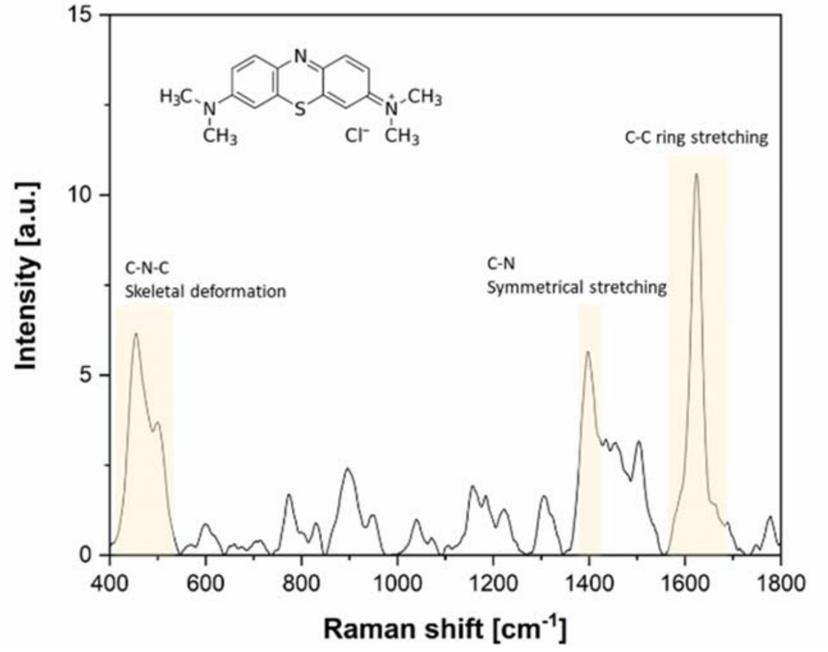

Figure S5: Raman spectrum of a solution of methylene blue 0.11M on PDMS. The most intense peaks and the corresponding vibrational modes are highlighted in the graph. The peak at 1623 cm^{-1} was used as a reference in the evaluation of the enhancement factor.

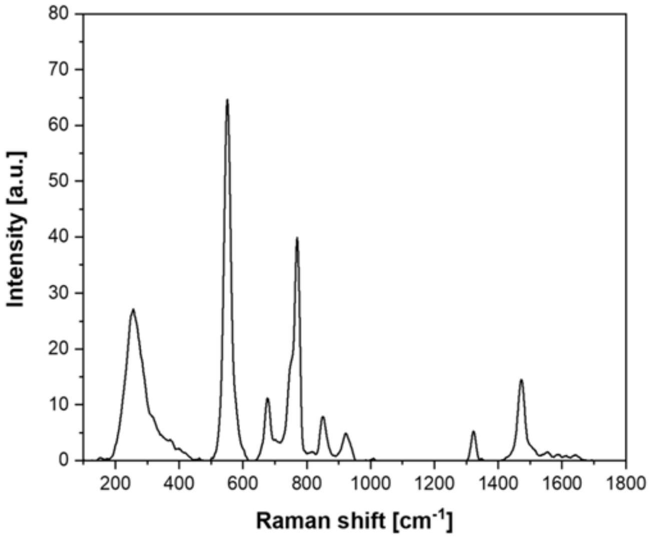

Figure S6: Raman spectrum of bare PDMS.

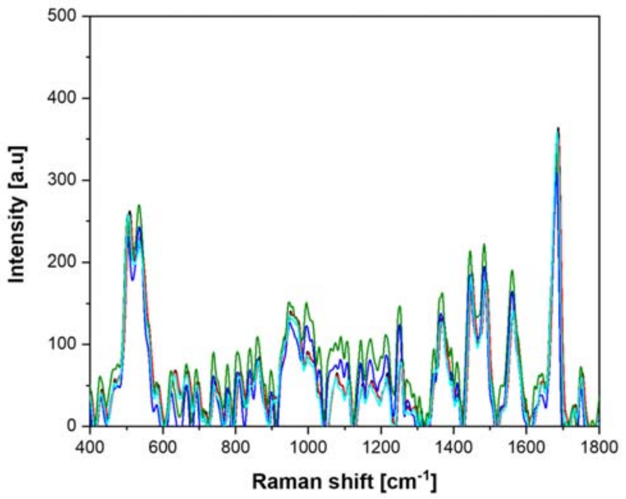

Figure S7: Raman spectra of 2.5·10^{-5} M methylene blue obtained on 5 points on the Ag NPs printed on PDMS. The printing conditions for the NPs are: writing speed of 60 μm/s, nozzle-substrate distance of 500 μm. Voltage/current settings used for the deposition V=1.3 kV and I= 10 mA.

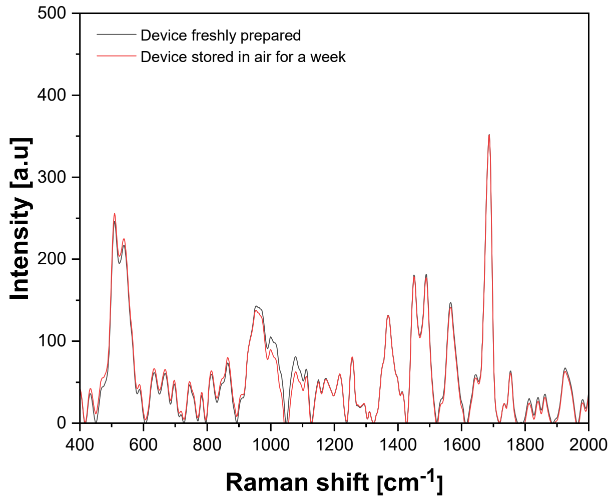

Figure S8: Comparison between the Raman spectra of 2.5·10^{-5} M methylene blue (MB) obtained by using a device with freshly printed Ag NPs patterns deposited in the chip chambers (in black) and with a device stored at ambient conditions for a week (in red).

6. **Tape test**

https://youtu.be/Jz2Hl2FV_Uo
